# Effectiveness of augmentative biological control depends on landscape context

**DOI:** 10.1038/s41598-019-45041-1

**Published:** 2019-06-17

**Authors:** Ricardo Perez-Alvarez, Brian A. Nault, Katja Poveda

**Affiliations:** 1 Department of Entomology, Cornell University, Ithaca, New York 14853 USA; 2 Department of Entomology, Cornell AgriTech, Cornell University, Geneva, New York 14456 USA

**Keywords:** Agroecology, Ecosystem services, Entomology

## Abstract

Biological pest control by natural enemies is an important component of sustainable crop production. Among biological control approaches, natural enemy augmentation is an effective alternative when naturally occurring enemies are not sufficiently abundant or effective. However, it remains unknown whether the effectiveness of augmentative biocontrol varies along gradients of landscape composition, and how the interactions with resident enemies may modulate the collective impact on pest suppression. By combining field and lab experiments, we evaluated how landscape composition influenced the effectiveness of predator augmentation, and the consequences on pest abundance, plant damage, and crop biomass. We show for the first time that the effectiveness of predator augmentation is landscape-dependent. In complex landscapes, with less cropland area, predator augmentation increased predation rates, reduced pest abundance and plant damage, and increased crop biomass. By contrast, predator releases in simple landscapes had a negative effect on pest control, increasing plant damage and reducing crop biomass. Results from the lab experiment further suggested that landscape simplification can lead to greater interference among predators, causing a decrease in predator foraging efficiency. Our results indicate that landscape composition influence the effectiveness of augmentative biocontrol by modulating interactions between the introduced predators and the local enemy community.

## Introduction

Agricultural intensification is one of the main drivers of biodiversity loss and landscape simplification^1^. Intensive agricultural practices simplify the landscape by decreasing crop species diversity and transforming natural habitats into more agricultural land. These changes in land-use patterns not only directly affect the diversity and composition of arthropod species^2^, but also potentially reduce the delivery of essential ecosystem services such as biological pest control^3^. As a consequence, farming systems have become increasingly reliant on synthetic inputs, which in turn exacerbate the negative effects of intensified agriculture on the environment and biodiversity conservation^4^. In this context, there is a strong need to promote farming practices that harmonize agricultural production with the conservation and sustainable use of biodiversity^5,6^.

Biological pest control by natural enemies has thus become an important component for sustainable crop production^7^. One strategy to improve biological control by resident natural enemies is enhancing habitat diversity through the provision of semi-natural vegetation in or around agricultural fields^8–10^. However, the most obvious potential disadvantage of these on-farm diversification strategies is that some land must be taken out of production, which may undermine any economic advantages gained through diversification^9,11^. Moreover, some of the benefits of habitat diversification may not manifest until a few years after implementation^12,13^. These drawbacks may discourage farmers from adopting this approach, particularly for high-value crops. An alternative and potentially complementary avenue to enhancing biological control is the release of mass-reared natural enemies in large numbers to obtain an immediate control of pests. In fact, augmentative releases of natural enemies have proven to be an environmentally and economically sound alternative to chemical pest control in a wide range of crop systems^14^.

Yet, few studies, if any, have evaluated the effects of enemy augmentation on pest control when other naturally occurring enemies are already present in the system. Theoretically, natural enemy augmentation could improve pest control through niche complementarity or facilitation among natural enemies^15,16^. Alternatively, increasing enemy abundance could disrupt pest control through intraguild predation and/or behavioral interference^17,18^. Due to the complexity of ecological interactions among natural enemies, a predictive framework is lacking for when increasing enemy abundance will strengthen or weaken pest suppression. Considering the paucity of knowledge on this issue, we evaluated the interactions among augmented and resident enemies and how such interactions affected pest control.

While practitioners have often focused on implementing conservation biological control on a field-scale, empirical and theoretical work have shown that the effectiveness of these local strategies can depend on the composition of the surrounding landscape^2,19–22^. For instance, habitat diversification practices, such as implementing flower strips and hedgerows, are more effective for enhancing biocontrol in moderately simple landscapes (i.e., dominated by agricultural areas) than complex landscapes (i.e., containing a high proportion of semi-natural habitats)^23,24^. Results from recent meta-analysis also found that agro-environmental practices had the greatest positive effect on cropland diversity and associated ecosystem services in simple landscapes^25–27^. Like habitat diversification, the effectiveness of augmentative releases of natural enemies to improve pest control may depend on the composition of the surrounding landscape. However, it remains to be seen whether the landscape-dependency patterns of augmentative biocontrol are comparable to those observed with other local management practices.

Landscape composition could moderate augmentative biocontrol effects through two different mechanisms. First, as predators and parasitoids generally benefit from semi-natural habitats^8,28^ (but see^29^), increasing landscape complexity can increase resident enemy diversity and abundance^30,31^. As a result, background levels of natural pest control can be sufficiently high in complex landscapes, making enemy augmentation ecologically redundant or even disruptive in this scenario (i.e., the intermediate landscape complexity hypothesis)^2,22^ (but see^32^). Second, higher levels of habitat heterogeneity that characterize complex landscapes can have positive effects on the ability of multiple enemies to coexist due to the presence of additional non-pest prey and greater range of microhabitats^33^. By providing conditions that dampen antagonistic interactions among natural enemies, increasing landscape complexity may lead to a net positive impact of enemy augmentation (i.e., the habitat heterogeneity hypothesis)^34^. Both hypotheses have experimental support in a variety of systems^23,35–37^; however, their validity for augmentative biocontrol practices remains unknown. Therefore, determining the landscape context under which enemy augmentation is likely to strengthen pest suppression is a key step towards developing ecologically-informed pest management strategies that benefit farmers. Furthermore, it is important to determine whether augmentative biocontrol might lead to cascading effects that influence plant performance (i.e., plant damage and crop biomass). Knowledge of crop productivity is important because this is the measure of biocontrol effectiveness of most relevance to growers, yet rarely quantified (but see^38–40^).

We addressed these questions using the interaction between cabbage crops (*Brassica oleracea* L. var. capitata), the lepidopteran pest complex (*Pieris rapae*, *Plutella xylostella*, and *Trichoplusia ni*), and its natural enemies. The lepidopteran complex is one of the most destructive pests of brassica crops worldwide, with annual management costs estimated in the billions of dollars^41,42^. In central New York (USA), a diverse community of naturally occurring enemies composed of 156 predator species and 7 parasitoid species is associated with the three primary lepidopteran pests of cabbage^43,44^. Among these natural enemies, two generalist predators have received considerable attention because they are common and relatively abundant in brassica crops in this region: the spined soldier bug, *Podisus maculiventris* (Hemiptera: Pentatomidae), and the convergent ladybird beetle, *Hippodamia convergens* (Coleoptera: Coccinellidae) (B. Nault, personal observation). However, natural densities of these predators are generally unable to reduce pest populations below damaging levels^45,46^, making augmentation of these commercially available predators a promising alternative to further increase the strength of pest supression. *Podisus maculiventris* preferentially feeds on lepidopteran larvae, whereas *H. convergens* feeds mostly on lepidopteran eggs^47^. Such differential predation on particular stages of the same prey may lead to complementarity among predators, and ultimately enhance biological control.

Here, we conducted field and laboratory experiments to evaluate how landscape composition influenced the effectiveness of augmentative biocontrol of lepidopteran pests by *P. maculiventris* and *H. convergens*, and the subsequent effect on plant damage and crop biomass. Specifically, we asked: 1. Does augmentative biocontrol effectively enhance pest control and reduce plant damage? 2. How does the interaction between landscape composition and enemy augmentation influence pest suppression? We experimentally addressed these questions by releasing predators in cabbage fields situated in landscapes of varying complexity and evaluating whether predator augmentation suppressed pest populations to a greater extent than resident natural enemies acting alone. We further explored potential mechanisms responsible for our field results by evaluating the independent and combined effect of *P. maculiventris* and *H. convergens* on pest predation in the laboratory.

## Results

### Relationship between the abundance of naturally occurring enemies and pest control

**Figure 1:**
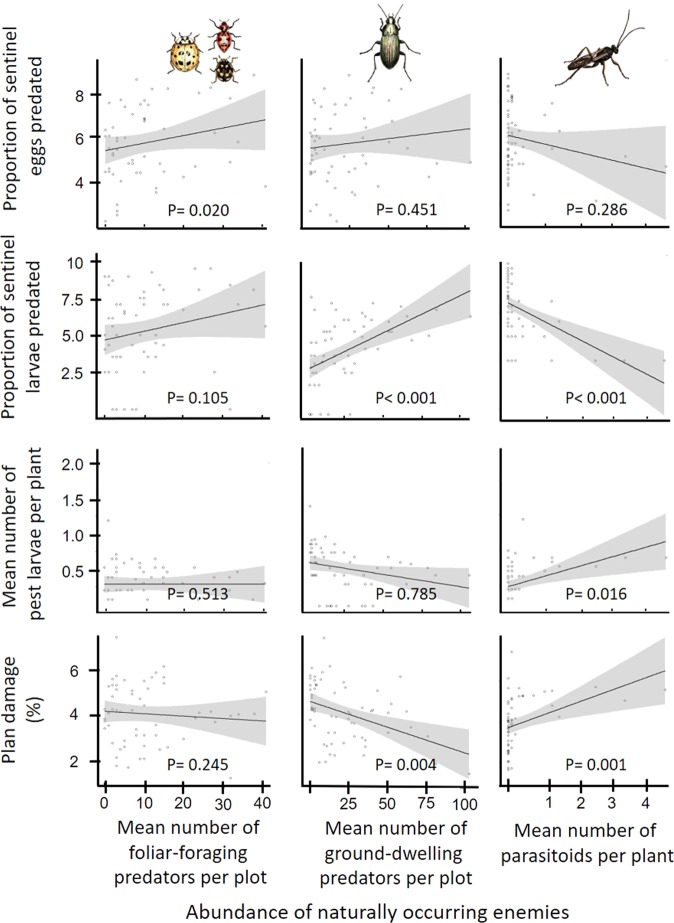
Relationships between the abundance of naturally occurring enemies and predation on sentinel eggs, predation on sentinel larvae, natural incidence of lepidopteran larvae, and plant damage. All response variables were square-root transformed. Lines are the fixed-effect predictions and associated 95% confidence intervals (gray shaded).

Predation on sentinel eggs was significantly associated with the abundance of foliar-foraging predators (F_1,45_ = 5.79, P = 0.020), whereas neither ground-dwelling predators (F_1,46_ = 0.578, P = 0.451) nor parasitoids (F_1,46_ = 1.166, P = 0.286) were significantly correlated with egg predation. In contrast to egg predation, larval predation did not correlate significantly with the abundance of foliar-foraging predators (F_1,47_ = 2.73, P = 0.105). Rather, larval predation was positively correlated with the total abundance of ground-dwelling predators (F_1,51_ = 22.02, P < 0.001), but negatively related to parasitoid abundance (F_1,48_ = 22.21, P < 0.001) (Fig. 1).

The natural incidence of *P. rapae* larvae was not influenced by either foliar-foraging (F_1,43_ = 0.435, P = 0.513) or ground-dwelling predators (F_1,47_ = 0.075, P = 0.785). However, there was a positive relationship between the abundance of *P. rapae* larvae and parasitoid abundance (F_1,44_ = 6.273, P = 0.016), suggesting that parasitoids were positively host density-dependent. Finally, plant damage was negatively influenced by the abundance of ground-dwelling predators (F_1,51_ = 9.134, P = 0.004), but positively correlated with parasitoid abundance (F_1,48_ = 11.55, P = 0.001). Foliar-foraging predators, on the other hand, had no effect on plant damage (F_1,47_ = 1.389, P = 0.245) (Fig. 1).

### Overall effects of augmentative predator releases

**Figure 2:**
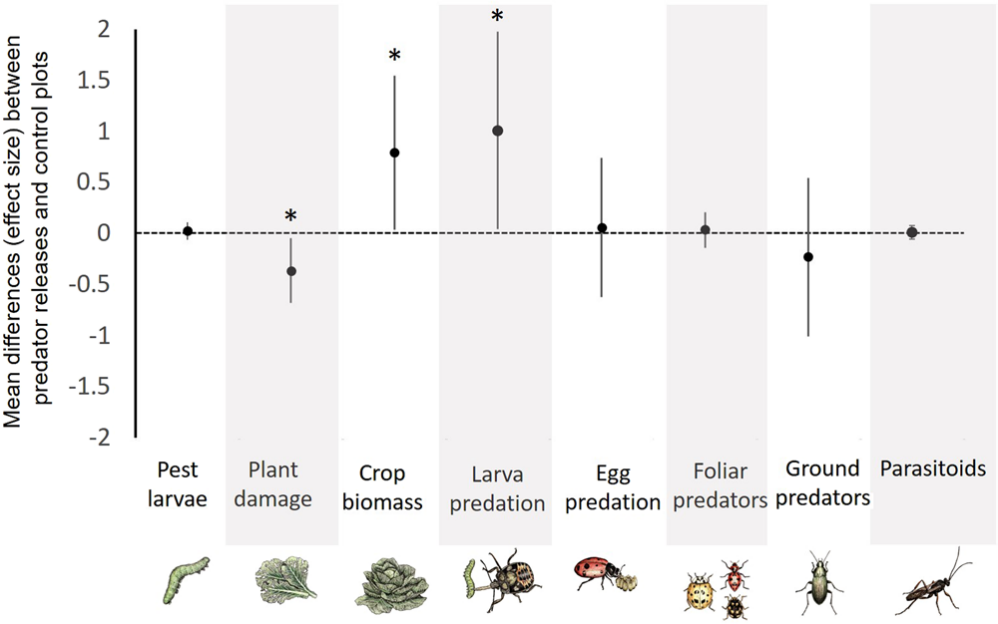
Effect sizes (mean ± 95% CI) for each response variable based on the difference in the marginal means between plots with and without augmentative predator releases. A positive effect size indicates that the mean of the predator release treatment is larger than the mean of control treatment, while a negative effect size indicates a higher control mean. Asterisks denote effect sizes that are significantly different from zero (P < 0.05). Summary statistics of the LMER and GLMM models used to estimate marginal means and confidence intervals are available in Table 1.

Augmentative releases of predators led to higher larval predation, lower plant damage, and higher crop biomass than the non-augmented control (Fig. 2, Table 1). Larval predation was 47% greater in the predator release treatment than in the control (t = 2.04, P = 0.047). Although larval predation was greater in the predator release treatment than in the control, we did not find differences in the mean abundance of naturally occurring caterpillars among treatments (t = −1.49, P = 0.137). Mean overall egg predation also did not differ significantly among predator and control plots (t = −0.27, P = 0.788). Lastly, no significant predator release effects were found for the overall abundance of any of the resident natural enemy groups (foliar-foraging: z = −0.37, P = 0.711; ground-dwelling: t = −0.03, P = 0.974; parasitoids: t = −0.54, P = 0.587).

**Table 1:** Statistical models for the effects of landscape composition and potential interactions with predator releases on lepidopteran larval abundance, plant damage, crop biomass, predation rates, and natural enemy abundance. Statistical models were used to estimate mean and 95% CI of effect sizes for overall effects of augmentative predator releases (Fig. 2) and the interactions with landscape composition (Figs 3–5). Dashed lines represent interaction terms not included in the final models because they were not significant (P > 0.05). Boldface text indicates significant relationships (P < 0.05).

Response variables and predictors	d.f.	F	P-value
**Lepidoptera abundance**			
Cropland (1000 m)	1, 11.887	4.885	**0.047**
Treatment (control and predator releases)	1, 40.407	5.200	**0.028**
Interaction (cropland × treatment)	1, 40.918	4.797	**0.034**
**Plant damage**			
Cropland (2000 m)	1, 11.335	7.656	**0.018**
Treatment (control and predator releases)	1, 43.651	19.520	**<0.001**
Interaction (cropland × treatment)	1, 43.617	23.079	**<0.001**
**Crop biomass**			
Cropland (2000 m)	1, 12.707	0.584	0.459
Treatment (control and predator releases)	1, 43.513	10.107	**0.003**
Interaction (cropland × treatment)	1, 43.049	10.976	**0.002**
**Predation on sentinel larvae**			
Semi-natural areas (2000 m)	1, 30.273	1.820	0.187
Treatment (control and predator releases)	1, 32.988	4.355	**0.045**
Interaction (semi-natural × treatment)	-----	-----	-----
**Predation on sentinel eggs**			
Semi-natural areas (2000 m)	1, 11.505	0.128	0.727
Treatment (control and predator releases)	1, 26.408	5.229	**0.030**
Interaction (semi-natural × treatment)	1, 25.844	4.618	**0.041**
**Ground-dwelling predators**			
Cropland (2000 m)	1, 11.301	6.753	**0.024**
Treatment (control and predator releases)	1, 31.629	0.001	0.973
Interaction (cropland × treatment)	-----	-----	-----
**Parasitoids**			
Semi-natural areas (1000 m)	1, 33.140	2.447	0.127
Treatment (control and predator releases)	1, 28.234	4.880	**0.035**
Interaction (semi-natural × treatment)	1, 27.759	6.105	**0.020**
**Foliar-foraging predators**		**z-value** ^**2**^	**P-value** ^**2**^
Semi-natural areas (500 m)		−0.791	0.429
Treatment (control and predator releases)		−5.121	**<0.001**
Interaction (semi-natural × treatment)		4.815	**<0.001**

Mean overall plant damage, estimated across the landscape complexity gradient, was reduced by 16% in the predator release plots relative to control plots (t = −2.28, P = 0.023). Importantly, average damage levels over the season were significantly correlated with the mean abundance of lepidopteran larvae (Pearson’s r = 0.33, P = 0.002), confirming that leaf-chewing caterpillars were largely responsible for the foliar damage observed in our field study. Moreover, overall crop biomass was 26% higher in the predator release plots compared with the control (t = 2.06, P = 0.040). Crop biomass was negatively correlated with plant damage such that plots with greater plant damage had overall lower crop biomass (Pearson’s r = −0.48, P = 0.033).

### Interactions with landscape composition

**Figure 3:**
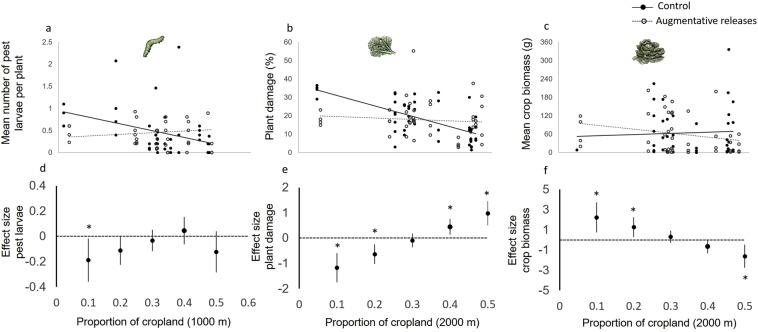
The effect of augmentative releases of predators on (**a**) lepidopteran larval abundance, (**b**) plant damage, and (**c**) crop biomass in landscapes of varying complexity. Predicted responses for the control (solid lines) and augmentative releases (dashed lines) treatments are calculated from the set of best supported linear mixed-effects models (lme4). Effects of the interactions between treatment and landscape complexity were significant in all cases (P < 0.05). In the top Figures (**a**–**c**) every point represents the mean treatment value in a given experimental plot for a given sampling period (i.e. 22 experimental plots and 4 sampling periods). The bottom figs. (**d**–**f**) are effect sizes (mean ± 95% CI) for lepidopteran larval abundance (**d**), plant damage (**e**) and crop biomass (**f**) based on the difference in the marginal means between plots with and without predator releases across the landscape complexity gradient. A positive effect size indicates that the mean of the predator plots is larger than the mean of control plots, while a negative effect size indicates a higher control mean. Pairwise comparisons were individually calculated at even intervals across the landscape complexity gradient. Asterisks denote effect sizes that are significantly different from zero (P < 0.05). Summary statistics of the LMER models used to estimate marginal means and confidence intervals are available in Table 1.

Local effects of predator releases on larval predation were influenced by the composition of the surrounding landscape (Fig. 3, Table 1). The abundance of lepidopteran larvae was significantly influenced by the interactive effect of predator releases and the proportion of cropland at the 1000-m scale (F = 4.80, P = 0.034; Fig. 3a). Similarly, plant damage and crop biomass were significantly influenced by the interactive effect of predator releases and the proportion of cropland at the 2000-m scale (plant damage: F = 23.08, P < 0.001; crop biomass: F = 10.98, P = 0.002; Fig. 3b,c). While caterpillar abundance and plant damage were significantly lower in the predator release treatment relative to the control in structurally complex landscapes (i.e., <20% cropland), this tendency was reversed in cropland-dominated landscapes (i.e., >40 cropland) (Fig. 3d,e). Crop biomass was also similarly affected by landscape composition with greater biomass in predator release treatments relative to control plots in complex landscapes, but in simple landscapes the opposite trend was observed (Fig. 3f).

**Figure 4:**
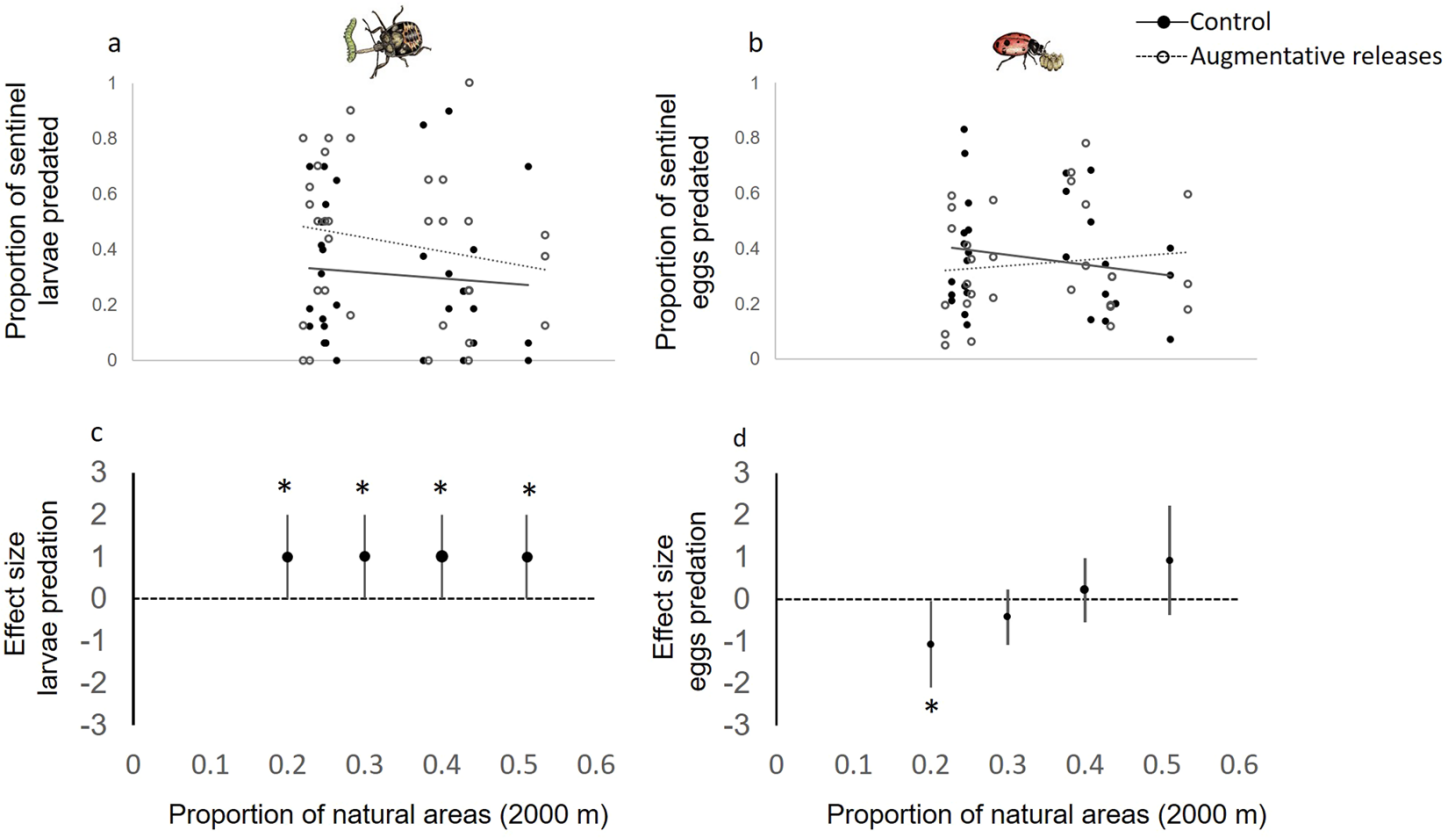
The effect of augmentative releases of predators on (**a**) predation on sentinel larvae, and (**b**) predation on sentinel eggs in landscapes of varying complexity. Predicted responses for the control (solid lines) and augmentative releases (dashed lines) treatments are calculated from the set of best supported linear mixed-effects models (lme4). Effects of the interactions between treatment and landscape complexity were significant (P < 0.05) for egg predation, but not for larvae predation. In the top Figures (a and b) every point represents the mean treatment value in a given experimental plot for a given sampling period (i.e. 22 experimental plots and 3 sampling periods). The bottom figs. (**c** and **d**) are effect sizes (mean ± 95% CI) for predation on sentinel larvae (**c**), and predation on sentinel eggs (**c**) based on the difference in the marginal means between plots with and without predator releases across the landscape complexity gradient. A positive effect size indicates greater predation rates in predator compared to control plots, while a negative effect size indicates lower predation rates in predator plots. Pairwise comparisons were individually calculated at even intervals across the landscape complexity gradient. Asterisks denote effect sizes that are significantly different from zero (P < 0.05). Summary statistics of the LMER models used to estimate marginal means and confidence intervals are available in Table 1.

The interaction between predator releases and landscape composition did not significantly affect predation of sentinel larvae (F = 0.02, P = 0.896; Fig. 4a, Table 1). As a result, larval predation was consistently higher in predator release plots irrespective of the landscape context (Fig. 4c). In contrast, egg predation was modulated by the interaction between predator releases and the proportion of semi-natural areas at the 2000-m scale (F = 4.62, P = 0.041; Fig. 4b). Predator releases increased egg predation in complex landscapes, but had no effect in simple landscapes (Fig. 4d).

**Figure 5:**
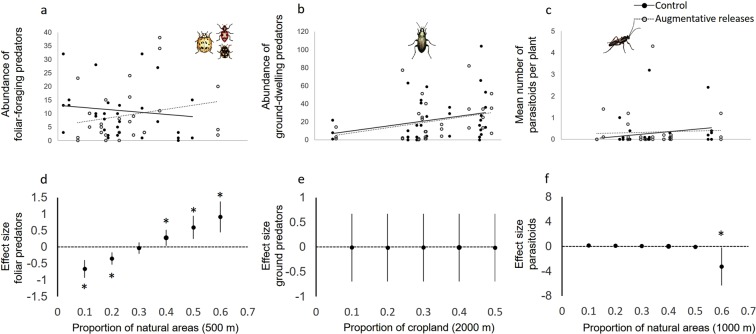
The effect of augmentative releases of predators on (**a**) foliar-foraging predator abundance, (**b**) ground-dwelling predator abundance, and (**c**) parasitoid abundance in landscapes of varying complexity. Predicted responses for the control (solid lines) and augmentative releases (dashed lines) treatments are calculated from the set of best supported linear and generalized mixed-effects models (lme4). Effects of the interactions between treatment and landscape complexity were significant (P < 0.05) for foliar-foraging predators and parasitoid abundance. In the top Figures (**a**–**c**) every point represents the mean treatment value in a given experimental plot for a given sampling period (i.e. 22 experimental plots and 3 sampling periods). The bottom figs. (**d**–**f**) are effect sizes (mean ± 95% CI) for foliar predators (**d**), ground predators(**e**), and parasitoid abundance (**f**) based on the difference in the marginal means between plots with and without predator releases across the landscape complexity gradient. A positive effect size indicates higher abundance of natural enemies in predator compared to control plots, while a negative effect size indicates lower abundance of natural enemies in predator plots. Pairwise comparisons were individually calculated at even intervals across the landscape complexity gradient. Asterisks denote effect sizes that are significantly different from zero (P < 0.05). Summary statistics of the LMER and GLMER models used to estimate marginal means and confidence intervals are available in Table 1.

Landscape composition also had strong effects on resident natural enemy abundance. Foliar-foraging predator abundance was best predicted by the interaction between predator releases and the proportion of semi-natural areas at the 500-m scale (z = 4.82, P < 0.001; Fig. 5a). Predator releases reduced the abundance of foliar-foraging predators in simple landscapes, but increased the abundance in complex landscapes (Fig. 5d). The activity of ground-dwelling predators was positively related to the proportion of cropland at 2000-m scale (F = 7.17, P = 0.021; Fig. 5b), but no difference was detected between predator release and control plots across the landscape gradient (Fig. 5e). Parasitoid abundance was influenced by the interactive effect of predator releases and the proportion of semi-natural areas at the 1000-m scale (F = 4.70, P = 0.037; Fig. 5c). However, contrary to foliar-foraging predators, predator releases had an adverse effect on parasitoid abundance in complex landscapes, but no effect in simple landscapes (Fig. 5f).

### Interaction between stinkbugs and ladybird beetles in the laboratory

**Figure 6:**
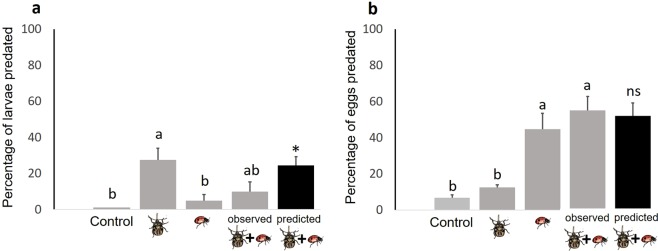
Predation rates (mean ± 1SE) on lepidopteran larvae (**a**) and eggs (**b**) under four treatments in the laboratory: (1) control, (2) stinkbugs alone, (3) ladybird beetles alone, and (4) stinkbugs with ladybird beetles. Different letters above bars indicate significant differences between treatments (two-way ANOVA followed by a Tukey HSD test, p < 0.05). Black bars represent predicted predation values for the combination of stinkbugs and ladybird beetles based on the multiplicative risk model^104^. The asterisk indicates a significant difference in predation between observed and predicted values (p < 0.05). Back-transformed means ± SE are presented but tests were performed using log-transformed data.

The outcome of the interaction between stinkbugs and ladybird beetles on prey predation depended on the developmental stage of the prey. Larval predation was greater in the stinkbug-only treatment (28%) than in the ladybird beetle-only treatment (5%), indicating that ladybird beetles played a smaller role in predating sentinel larvae compared with stinkbugs (F_3,28_ = 7.78, P < 0.001; Fig. 6a). Further, the combined effect of stinkbugs and ladybird beetles on larval predation (10%) was not significantly different from the effect of stinkbugs alone. However, there was a significant difference between observed and predicted larval predation in the combined natural enemy treatment (F_1,14_ = 5.14, P = 0.040), indicating an antagonistic interaction between stinkbugs and ladybird beetles. Total larval predation declined 64% in the presence of both predators relative to stinkbugs alone. Thus, larval predation by stinkbugs was constrained by antagonistic interactions with ladybird beetles.

In contrast to results with larval predation, egg predation was significantly higher in the presence of ladybird beetles than in treatments without them, while egg predation by stinkbugs was negligible (F_3,28_ = 7.78, P < 0.001; Fig. 6b). However, unlike larval predation, the combination of predators neither strengthened nor weakened egg predation. The observed combined effect of stinkbugs and ladybird beetles on egg predation was not significantly different from those predicted based on the sum of each individual predator effect (F_1,14_ = 0.07, P = 0.794), suggesting that these predators had an additive effect on egg predation.

## Discussion

We demonstrated that the local effectiveness of predator augmentation is moderated by the composition of the surrounding landscape. Indeed, predator releases had positive trophic cascading effects that increased predation rates, reduced pest abundance and plant damage, and increased crop biomass in complex landscapes. In contrast in simple landscapes, predator releases had a negative effect on pest control, increasing plant damage and reducing crop biomass. Thus, the interaction between local augmentative biocontrol and landscape composition not only influenced the intermediate ecosystem service of pest control, but also had downstream consequences at the crop production level. Importantly, neglecting the landscape-mediated effects on the efficacy of predator augmentation may lead to inconsistent and misleading outcomes, which ultimately has consequences for growers who wish to implement this practice. While we recognize the potential implications of our findings for the management of lepidopteran pests in the cabbage system, our discussion here focuses on identifying the ecological mechanisms underlying the variation in the effectiveness of augmentative strategies. Knowledge of these mechanisms is key to increasing our ability to predict and understand when enemy augmentation can lead to net positive effects on pest control in a wide range of cropping systems.

### Landscape effects on naturally occurring enemies

Previous work has illustrated the importance of naturally occurring predators and parasitoids for lepidopteran pest suppression at the field scale^43,44^. Here, we build on those studies by showing that the abundance of naturally occurring enemies are directly influenced by the composition of the landscape surrounding our focal fields. Simple landscapes, defined as landscapes with high proportions of cropland, were positively correlated with the abundance of foliar and ground-dwelling predators (based on the control plots). In contrast to predators, parasitoids were far less abundant in simple landscapes. These results indicate that the relative contribution of different naturally occurring enemies to pest suppression varies across the landscape complexity gradient, as reported elsewhere^16,48^. On one hand, parasitoids were positively host density-dependent (i.e., positive relationship between host and parasitoid abundance), but by themselves were not capable of lowering pest abundance and concomitant plant damage. Ground beetles, on the other hand, showed stronger positive impacts on larvae biocontrol with subsequent reductions in plant damage particularly in simple landscapes, but their densities did not respond numerically to changes in pest density. Naturally occurring coccinellids showed no clear contribution in reducing densities of pest larvae or plant damage, but they were positively associated with egg predation.

### Interaction between landscape composition and predator augmentation

Although our findings suggest that naturally occurring enemies can contribute to the regulation of *P. rapae* populations, their control levels varied significantly over the course of the growing season and among landscapes. Therefore, complementary strategies are desirable to achieve stable and economic pest control. Results from our study suggest that augmentative releases of predators have the potential to supplement the strength of pest control provided by naturally occurring enemies under certain ecological contexts. Over the course of our study, predation on sentinel larvae was consistently higher at sites supplemented with predators when compared with predation in control plots. Yet, predator augmentation failed to provide consistent control of naturally occurring pest larvae across sites, which is presumably tied to differences in landscape composition. While previous studies have identified a number of ecological mechanisms that may limit the effectiveness of augmentative biological control in the field (e.g., climatic constraints, release timing and release rates, quality control)^49–51^, this is the first study highlighting the importance of landscape context in mediating the effectiveness of enemy augmentation as a pest management strategy.

Several non-mutually exclusive mechanisms could explain the landscape-moderated effectiveness of predator augmentation on pest control reported here: (1) functional complementarity among augmented and resident enemies in complex landscapes, (2) antagonistic interactions (i.e., intraguild predation and predator interference) among enemy species in simple landscapes, and (3) via landscape-mediated changes in the composition of the local enemy assemblage, which in turn may determine the sign and strength of interactions with the augmented predators.

First, landscape complexity can enhance the complementarity among augmented and resident enemies, and thereby the strength of pest suppression^16^. Complex landscapes containing large amounts of semi-natural habitats can provide natural enemies with alternative food sources and suitable microhabitats that together might favor the coexistence of species with overlapping feeding niches^52^. Indeed, habitat heterogeneity has been positively linked to reductions in antagonistic interactions among natural enemies, thus increasing overall pest control^33,35,53^. Our results support the idea that increasing enemy abundance may have net positive effects on pest control and plant performance, but only in complex landscapes where habitat heterogeneity may create favorable conditions for complementarity between augmented and resident enemies.

Second, our results also provide empirical support to the notion that landscape simplification potentially increase antagonistic interactions among natural enemies by reducing the diversity of habitats that provide key foraging and nesting resources enabling species coexistence. The role of antagonism among natural enemies in the outcome of biological control can be particularly important in situations when generalist predators are released. For example, *P. maculiventris*, although thought to be an effective biocontrol agent of lepidopteran pests^54–56^, can also potentially feed on other natural enemies of *P. rapae*, including predatory coccinellids and hymenopteran parasitoids^57–60^. However, the extent to which increasing the abundance of *P. maculiventris* may lead to decreases in other natural enemies under field conditions was not reported prior to this study. In our study, the abundance of naturally occurring coccinellids decreased significantly following the introduction of *P. maculiventris* in simple landscapes. It is conceivable that some of the reduction in coccinellid abundance was due to the increase in dispersal from the experimental plots supplemented with *P. maculiventris* rather than actual predation. In line with this finding, Moran & Hurd (1994)^61^ recorded increased emigration rates of naturally occurring spiders in response to increased density of mantid predators. Regardless of the causal mechanism, increasing the abundance of *P. maculiventris* through augmentative releases in simple landscapes can negatively affect other predators, thereby reducing overall pest control. Although *P. maculiventris* also reduced parasitoid abundance in complex landscapes, this effect did not disrupt overall control of *P. rapae* because parasitoids only occurred when pest densities were already high.

Third, we showed that predators can disrupt one another via non-trophic interactions in a controlled laboratory experiment, which was designed to mimick a simple landscape. The effectiveness of *P. maculiventris* in feeding on *P. rapae* larvae was numerically reduced in the presence of *H. convergens*, as compared with *P. maculiventris* acting alone. Thus, our laboratory experiment results were consistent with our field findings of reduced biocontrol of *P. rapae* in simple landscapes. Factors leading to reduced effectiveness of *P. maculiventris* in simplified landscapes may have included changes in predator or prey foraging activity induced by the presence of other predators (i.e., *H. convergens*)^62,63^. Recent studies have shown that such behavioral effects are ubiquitous in biocontrol systems and potentially affect pest suppression^64–68^, as demonstrated herein. In complex landscapes, some of the mechanisms of reducing niche overlap (e.g., spatial separation or the availability of alternative preys) must be acting to maintain the effectiveness of *P. maculivestris* despite the potential interference with other natural enemies. These results underscore the importance of considering non-trophic interactions (e.g., predator interference) in concert with intraguild predation when interpreting the outcomes of multiple-predator effects.

Finally, the landscape context may influence the effectiveness of augmentative biocontrol via changes in the composition of the naturally occurring enemies. Unlike our finding that augmentation effectiveness was inversely related to habitat simplification, augmentation of natural enemies has been used successfully for decades in greenhouses^69^, even though enclosed environments are arguably simpler than open-field crops. This counterexample suggests that factors other than habitat complexity can, in some cases, determine whether positive effects of predator augmentation are realized within diverse enemy communities. Compared with open field crops, greenhouses virtually lack any naturally occurring enemies that could potentially interfere with the released agent. In fact, species richness and composition are important determinants of the range and direction of interactions among natural enemies^70^, especially in open field crops where enemy communities, even in simplified landscapes, are more complex and diverse than those of greenhouses^71^. Because there is considerable variation in the responses of different enemy taxa to changes in landscape composition, it follows that predator augmentation effects may vary in response to shifts in the identities of the species present in the local community. Naturally occurring enemies may potentially disrupt augmented predators either directly through mutual interference or intraguild predation, or indirectly via reduction in prey densities thorough pest consumption. Therefore, the effectiveness of enemy augmentation is not determined solely by the landscape context, but by how the local enemy assemblage interacts with the augmented enemies. Such context-dependency in the interaction among enemies hinders the formation of general rules to predict the net effects of predator augmentation across systems. Our study, nevertheless, provides new insights into the mechanisms whereby the combination of augmented and resident enemies may be expected to enhance pest control, and thereby offer a conceptual framework to make plausible predictions that are amenable to further testing in other systems.

Taken together, our work clearly demonstrates that the benefits of natural enemy augmentation are landscape-dependent. As such, our work adds to a growing set of evidence that biological pest control is not simply a function of enemy diversity and abundance, but also the landscape context in which enemies interact^72,73^. Fortunately, some general rules of these landscape dependency patterns have started to emerge to provide instructive management of certain landscape contexts where local agricultural practices may be more likely to enhance biological control. For example, planting flower strips adjacent to crop fields tends to produce large effects on boosting natural enemy populations in simple landscapes, but reduced impacts in complex landscapes^23^. Other agri‐environmental schemes aimed at pest control also have been shown to be more effective in simple than complex landscapes^37,48^. However, our study found landscape dependency patterns that differ from those described above, indicating that more research on augmentation practices is needed before broader conclusions can be drawn. For example, it would be important to verify the consistency of our results over multiple cropping seasons. Also, studies in other cropping systems and geographic regions are important to test the generality of our findings.

## Conclusions

Augmentative biocontrol has long been recognized as a promising pest control alternative to conventional pesticide use when used as part of a comprehensive integrative pest managment approach. However, the effectiveness of augmentative biocontrol to manage agricultural pests in field situations has been questioned because they have mixed records of success. Our research expands on previous work exploring the ecological factors associated with such conflicting outcomes^49,74^ by demonstrating that the effectiveness of augmentation depends strongly on the composition of the surrounding landscape. In the context of our study region, augmentative biocontrol was more effective in suppressing lepidopteran pests in complex than in simple landscapes. Clearly, these results are system-dependent and the specifics arising from other enemy-pest systems can create idiosyncrasies that demand case-by-case consideration. For example, a different conclusion might be reached by considering other natural enemies (e.g., augmentation of specialist parasitoids) or different target pests (e.g., aphids and flea beetles). From an applied perspective, this context dependency can be frustrating, but it must be acknowledged if we hope to effectively integrate natural enemy augmentation strategies in agricultural production systems. To this end, we need to move beyond the debate concerning the merits of using multiple vs. single species introductions of natural enemies with little regard for the spatial patterns in agricultural landscapes^75–77^. Ultimately, a greater understanding of landscape-moderated interactions between pests and their natural enemies would provide much needed information for pest management practitioners with respect to how and where natural enemy augmentation can be implemented more effectively.

## Materials and Methods

### Study region

The study was carried out from June to October 2015 in the Finger Lakes Region (42°26′N, 76°30′W) of New York State, USA. The landscape in this region is characterized by a mosaic of cropland and semi-natural habitats. Cropland in these landscapes mainly consisted of corn, soybean, winter wheat and crucifers, while semi-natural areas are composed of shrublands, woody wetlands, and mixed forest. We selected 11 farms across the study area to encompass a gradient of landscape complexity from landscapes with large amounts of semi-natural habitats (2% cropland) to simple landscapes dominated by crops (50% cropland) within a 1000 m radius around each farm. All farms selected for the study were either organic or used minimal inputs for pest management.

To quantify the landscape composition surrounding each farm, the proportion of semi-natural areas and cropland were calculated at three scales: 500 m, 1000 m, and 2000 m. These spatial scales are suitable for analyzing the effects of landscape context on pest control and natural enemies^21^. The landscape was characterized using the 2015 National Agricultural Statistics Service Cropland Data Layer for New York^78^ in ArcGIS 10.1.

### Experimental plots

Seeds of fresh-market cabbage (*B. oleracea* var. capitata cv. Capture) were grown in an organic potting mix (sunshine®, Sun Gro Horticulture Inc., Bellevue, WA, USA), and fertilized with organic fish fertilizer 2-4-1(N-P-K) (Neptune’s Harvest®, Gloucester, MA, USA) for seven weeks under greenhouse conditions. Plants were eight weeks old when they were transplanted to the field.

On each of the 11 farms, we established two experimental plots. One plot was randomly chosen for the augmentative predator release treatment while the other served as a non-release control. Plots within the same farm were separated by 334 ± 41 m (mean ± 1 SE), and the mean distance between farms was 7.2 ± 2.3 km. Care was taken to minimize fine-scale landscape heterogeneity between experimental plots within the same farm. Plots within the same farm primarily differed in the predator release treatment, while landscape context, plot size and shape, and abiotic conditions were similar for each pair.

Each experimental plot consisted of ten 7.2-m rows, with 15 cabbage plants per row. Row and plant spacing were 0.9 m and 0.45 m, respectively. Plants were transplanted across farms over two consecutive weeks in mid-June 2015. Experimental fields within the same farm were planted on the same day. Plants were fertilized during transplanting and again one month later using 8-3-3 (N-P-K) granular compost at a rate of 5 kg/100 m^2^ (Kreher’s® composted poultry manure, Clarence, NY, USA). All experimental plots were managed without fungicides or insecticides, and weeds were removed at two-week intervals.

### Augmentative releases of predators

The predator release treatment included both *Podisus maculiventris* nymphs and *Hippodamia. convergens* adults. Both the nymphal and adult stinkbugs display high predation rates on lepidopteran larvae, so we released fourth and fifth instars in our experiments to minimize dispersal after release and increase the potential for season-long pest control. Ladybird beetle larvae were not available commercially, which precluded us from using less-mobile stages. Predators were released three times throughout the season at the seedling, pre-cupping, and early head formation growth stages^79^. Releases were conducted early in the season, as previous studies have shown that early control is key to the success of biocontrol strategies in field settings^80,81^. Approximately 200 stinkbugs and 600 ladybeetles were released per plot each time by carefully deploying them on the leaves. These release rates equaled 1.3 sting bug nymph/plant and 4 ladybird adults/plant. These are commonly recommended release rates by commercial vendors^82–85^. No predators were released in the control plots.

*P. maculiventris* were obtained from eggs purchased from a commercial supplier (Beneficial Insectary Inc., Redding, CA, USA) and reared on a diet of mealworms, *Tenebrio molitor* (L.), and cabbage seedlings. The stinkbug colony was kept at 25.5 ± 2.0 °C, 60% RH, and a photoperiod of 16:8 (L:D) following the methods of De Clercq *et al*.^86^. Adults of *H. convergens* were obtained from a commercial supplier (Arbico Organics, Oro Valley, AZ, USA). Ladybird beetles were stored at 7 °C until we released them in the field.

### Measuring predation rates in the field

Predation rates provided by resident and augmented predators were quantified using *Trichoplusia ni larvae* and eggs as sentinel prey. *Trichopluisia ni* were commercially available and easier to manipulate in field studies than *P. rapae*. *Trichopluia ni* larvae and eggs were obtained from a commercial insectary (Benzon Research Inc., Carlisle, PA, USA). For estimating larval predation, 5 third-instars (13.6 ± 0.23 mm long) were placed on the upper leaves of four randomly selected plants per plot (i.e., 20 larvae per plot). After 24 h of exposure in the field, the remaining larvae were counted to determine the number of larvae consumed by predators. Larvae were considered predated if they were completely missing, or showed evidence of predation such as necrotic tissue around an open wound.

To estimate egg predation per plot, paper discs containing approximately 30 *T.ni* eggs (range: 19–76) were fixed to the underside of 10 × 10 cm pieces of corrugated plastic board (Coroplast®, Vanceburg, KY, USA) that provided a standardized foraging platform for predators. Five egg platforms were positioned at crop height and placed between the leaves of the plants where sentinel larvae were deployed. All egg masses were inspected after 24 h, and the number of eggs remaining were counted to determine predation rates. Eggs were considered predated if they were missing, the chorion presented clear evidence of attack (i.e., chewing predator), or the contents of the egg had vanished (i.e., attacks by a piercing-sucking predator). To distinguish sentinel prey predation from unknown losses due to handling and rainfall, we enclosed one plant per site in a cage that excluded natural enemies. Cages consisted of a 0.2 × 0.2 × 0.2 m^3^ mesh plastic screen (BioQuip, Rancho Dominguez, CA, USA) with openings of 1.1 × 0.7 mm^2^, and whose bottom edges were buried 5 cm into the ground. Plants in these cages were infested with sentinel prey in the same fashion as the uncaged plants. Net mortality due to predation was determined by assessing mortality from uncaged plants and subtracting it from mortality from caged plants.

We repeated the sentinel prey experiment three times per plot at the seedling, pre-cupping, and early head formation growth stages. Thus, we had three temporally separated dates that allowed us to account for the temporal differences in predation rates throughout the season.

### Sampling of lepidopteran pests and their natural enemies

To assess lepidopteran abundance, plants were visually inspected for larvae during the seedling, pre-cupping, early head formation, and maturation growth stages^79^. In each plot, ten randomly selected plants were destructively sampled and the number of larvae were recorded on each plant. To avoid possible edge effects, plants within 1 m of the edge of the plot were not sampled. A total of 294 caterpillars were collected in the experimental plots, with *P. rapae* as the dominant species (94% of the total caterpillars collected) followed by *P. xyllostela* (5%) and *T. ni* (0.4%).

Naturally occurring predators and parasitoids were sampled using yellow sticky cards, pitfall traps, and visually inspected plants. Natural enemies from these samples were categorized into functional groups as foliar-foraging predators, ground-dwelling predators, and parasitoids. Our analysis was restricted to species known to attack either lepidopteran eggs or larvae based on previous observational and experimental studies (e.g.^43,87^). For foliar-foraging predators, we focused on the three dominant species of coccinellids in our system: the native *Coleomegilla maculata* and the two-exotic species, *Harmonia axyridis* and *Propylea quatuordecimpunctata*. Abundance of all three coccinellid species were pooled to obtain the overall abundance of relevant foliar-foraging predators for each plot. For ground-dwelling predators, carabid beetles were collected and identified to species. Following identification, we gathered information from the literature to further classify carabids into three diet categories: carnivorous, omnivorous or phytophagous^88,89^. Only carnivorous species were kept in further analyses. Altogether 25 predatory carabid species were collected, of which three species (*Bembidion quadrimaculatum*, *Poecilus chalcites* and *Poecilus lucublandus*) made up 66% of the total capture. As with coccinellids, the abundance of predatory carabids for each plot was pooled in subsequent analysis. Lastly, we measured parasitoid abundance by focusing our sample efforts on *Cotesia rubecula* (Hymenoptera: Braconidae), the most important specialist parasitoid of *P. rapae* larvae in the study region^90^. The parasitoids of the *T. ni* and *P. xylostella* were not investigated because both pests occurred in small numbers in our system (i.e. <6% of the total caterpillars collected).

Sampling for all natural enemies was conducted three times during the season at the seedling, pre-cupping, and early head formation stages. Foliar-foraging predators, ground-dwelling predators and parasitoids were sampled using sticky cards, pitfall traps and visual inspection of plants, respectively. On each sampling time, one sticky card (15 × 30 cm, BioQuip, Rancho Dominguez, CA, USA) was positioned at crop height in the center of each plot. The sticky cards were retrieved after 15 days and the number of foliar-foraging predators were recorded. For the pitfall traps, a 540 mL clear plastic cup (9 cm diameter openings, Fabri-kal corp., Kalamazoo, MI, USA), was filled with a mixture of water and a few drops of organic, odorless detergent (Dr. Bronner’s Unscented Pure Castile Soap, Vista, CA, USA). A total of five traps were placed within the rows between cabbage plants; four traps were located near the corners and one in the center row of the plot. Each trap was protected from rain and direct sunlight by a plastic plate (15 cm in diameter) held approximately 10 cm above the trap. Pitfall traps were collected after 24 h and the number of ground-dwelling predators was recorded. Finally, on each sampling date, parasitoid abundance was estimated by counting the total number of parasitoid cocoons (i.e, pupa) on ten randomly selected plants per experimental plot. Parasitoids were identified using diagnostic morphological characters described by Van Driesche (2008)^91^.

### Plant damage and crop biomass

Insect damage and crop biomass were assessed from the same ten plants used for lepidopteran censuses at four sampling times during the season. Damage was quantified using a modified version of the method of Lim *et al*.^92^, where a plant is classified into one of the following eight categories based on the percentage of leaf area removed: 0, 5–10,10–20, 20–40, 40–60, 60–80, 80–100, or 100%). Visual estimates of damage provide the fastest and most cost-effective method for quantifying herbivory^93^, and previous studies have shown they can be precise and accurate to estimate economic thresholds for lepidopteran defoliation in cabbage^94^. For analysis, we assumed the estimated proportion of damage on each plant to be the median of each category (0, 7, 15, 30, 50, 70, 90, 100, respectively). Crop biomass was determined by weighing the plants after they had been oven-dried at 60 °C for 7 days. Although the crop biomass at the end of the season (i.e. maturation growth stage) is a measure of crop yield (i.e. marketable cabbage head weight), we used the crop biomass throughout the season in our analyses rather than only final biomass, as the former allowed us to account for the temporal differences in the effectiveness of augmentative biocontrol. Analysis using only final crop yield produced qualitative similar results (Supplementary Fig. S1).

### Laboratory experiment

Controlled lab experiments were conducted to quantify the individual and combined effect of stinkbugs and ladybird beetles on pest predation, independently from the effects of landscape context. Experimental units were 28 × 28 × 28 cm cages, covered on all sides, expect the bottom, with a mesh screen opening of 1.1 × 0.7 mm (BioQuip, Rancho Dominguez, CA, USA). Each experimental unit consisted of a single potted cabbage plant (*B. oleracea* var. capitata cv. Capture) with six fully-expanded true leaves. To begin the experiment, all cages received 5 third-instar larvae and one egg mass (approximately 30 eggs) in the same fashion as the sentinel field experiment. *T. ni* larvae were allowed to establish for 1 h before predator introduction.

Predators were released into individual cages according to four treatments: control (no predators added), stinkbug treatment (2 fifth-instars added), ladybird beetle treatment (5 adults added), and the interaction treatment (2 fifth-instar stinkbugs and 5 ladybird beetles adults added). These densities were chosen because they mimicked those used in the sentinel field experiment. Each treatment was replicated eight times. The experiment had an additive design (i.e., overall predator density is higher in the multi-species treatment compared to the single-predator treatment), because this approach better reflects the effect of predator augmentation. Predators were starved for 24 h before being introduced to standardize hunger levels across treatments and then allowed to feed for an additional 24 h, after which the number of larvae and eggs remaining in the cages were recorded. The experiments were conducted at 25 ± 2 ◦C, 60 ± 5% RH and a 14:10 (L:D) h photoperiod.

### Statistical analysis

To analyze the direct effect of the abundance of naturally occurring enemies on biocontrol of sentinel prey, pest incidence, and plant damage, we used linear mixed-effect models in R with the nlme package^95^. Abundances were averaged for each functional group separately (i.e. foliar-foraging predators, ground-dwelling predators, and parasitoids) and for each sampling period. Response variables were square-root-transformed to meet assumptions of normality and homoscedasticity^96^. For all models, we also included farm as random effect to account for other potential sources of variability associated with each geographic location (e.g., environmental or management intensity differences). Statistical significance of the abundance of each functional group was assessed by conditional *F*-tests^97^.

The effects of landscape complexity and potential interactions with predator releases, on lepidopteran pest abundance, natural enemy abundance, predation rates, plant damage, and crop biomass, were examined using linear (lmer) and generalized linear mixed-effect models (glmer)^98^. Fixed factors in the models included treatment (with or without predator releases), landscape complexity, and the treatment by landscape interaction. Landscape complexity was defined as either the proportion of cropland or the proportion of semi-natural areas as both variables were highly correlated at all scales (Spearman’s r_s_ < −0.45, P < 0.001 at all scales). Random effects in all models included farm and sampling time to account for the crossed experimental design (i.e., each plot was measured on multiple dates and multiple plots were measured on each date). Response variables were square-root transformed prior to analysis to meet normality assumptions and avoid heteroscedasticity. Assumptions were checked according to the graphical validation procedures recommended by Zuur *et al*.^96^. Models for foliar-foraging predators did not meet distributional assumptions, and therefore were analyzed using a generalized linear mixed model with a Poisson error distribution. Model simplification was done using a backwards-stepwise selection (lmer) or likelihood ratio tests (glmer) based on Akaike’s Information Criterion (AIC) where non-significant predictors were removed (P > 0.05). We assessed the statistical significance of fixed effects and interaction terms by F-tests based on the Satterthwaite approximation (lmer) or Wald Z-test (glmer)^99,100^. Separate models were fitted for each landscape scale (i.e., 500, 1000, and 2000 m), and the scale with the highest explanatory power for each response variable was determined by comparing the AIC values of the minimum adequate models^101^. The most predictive scale for each response variable was then used in further analyses. Subsets of best models for each response variable are provided in Supplementary Table S1. Mantel test^102^ indicated no spatial autocorrelation in the residuals of the final models (Supplementary Table S2).

To better understand potential differences between the predator release treatment and the control, we used the final models to estimate the marginal means and 95% confidence intervals for each response variable with the “emmeans” package in R^103^. For all response variables (i.e., lepidopteran abundance, predation rates, plant damage, crop biomass, and natural enemy abundance), we used preplanned contrast to determine whether mean differences between plots with and without predator releases (i.e, effect size) were significant. We first estimated the mean effect size across the entire landscape complexity gradient to get an overall quantitative assessment of the consequences of predator augmentation for each response variable. In a second group of comparisons, we estimated the effect size of each response at even intervals over the landscape complexity gradient (range: 0–0.6) to test the hypothesis that the effects of augmented predators were contingent on the characteristics of the surrounding landscape. Pairwise multiple comparisons were calculated using the Bonferroni correction for an overall error rate of 0.05. Comparisons were conducted using the emmeans package.

For the laboratory experiment, we examined predation rates on lepidopteran larvae and eggs using a two-way ANOVA followed by a Tukey HSD test at P < 0.05 including the factors: stinkbugs (with or without), ladybird beetles (with or without), and their interaction. Predation rates were log-transformed to meet the assumptions of the analysis. To further examine these data, we used a multiplicative risk model^104^ followed by ANOVA comparing the expected and actual predation rate values to assess whether combined predators act independently (i.e., observed and predicted values do not differ, so that its combined effect is additive), antagonistically (i.e., observed values are less than the predicted values), or synergistically (i.e., observed values exceed the predicted values) on prey populations^60^. All statistical analyses were done using R v. 3.2.3^105^.

## Supplementary information

Supplementary Information-Landscape effects on augmentative biocontrol

## Data Availability

The datasets generated during and/or analysed during the current study are available from the corresponding author on reasonable request.
