# Supplementary material for: Effectiveness of augmentative biological control depends on landscape context
Source: Sci Rep. 2019 Jun 17;9:8664. doi: 10.1038/s41598-019-45041-1 (PMC6572857; doi:10.1038/s41598-019-45041-1)
Supplement: Supplementary file 1 — Supplementary Information-Landscape effects on augmentative biocontrol [file 41598_2019_45041_MOESM1_ESM.pdf]

Supplementary Information

Perez-Alvarez, R., Nault, B. & Poveda, K. Effectiveness of augmentative biological control depends on landscape context (2019).

Supplementary tables S1, S2, & S3

Supplementary Fig. S1.

Supplementary Table S1. Model selection for effects of landscape composition and potential interactions with predator releases on lepidopteran larval abundance, plant damage, crop biomass, predation rates, and natural enemy abundance.

Supplementary Table S2. Results of Mantel test of spatial autocorrelation in the residuals of the final models.

Supplementary Table S3. Statistical models for the effects of landscape composition and potential interactions with predator releases on both final crop yield quality and crop yield quantity.

Supplementary Fig. S1. The effect of augmentative releases of predators on final crop yield based on quality and quantity measures in landscapes of varying complexity.

Supplementary Table S1. Model selection for landscape effects and potential interactions with predator releases on lepidopteran larval abundance, plant damage, crop biomass, predation rates, and natural enemy abundance. The overall best model (most parsimonious) and competing models are presented. The overall best models are bolded. The AICc values, AICc difference (ΔAICc) and conditional coefficient of determination[1] (R^2) are given for each model. Models were selected based on second order Akaike Information Criterion (AICc).

Response variable	Landscape variable	Scale (m)	Model	AICc	ΔAIC_C	R^2
Lepidoptera abundance	Cropland	500	Intercept + Cropland + Treatment + Cropland *Treatment	53.645	3.322	0.733
	**Cropland**	**1000**	**Intercept + Cropland + Treatment + Cropland *Treatment**	**50.322**	**0**	**0.766**
	Cropland	2000	Intercept + Cropland + Treatment + Cropland *Treatment	51.818	1.496	0.760
	Semi-natural areas	500	Intercept + Semi-natural+ Treatment + Semi-natural*Treatment	55.563	5.241	0.795
	Semi-natural areas	1000	Intercept + Semi-natural+ Treatment + Semi-natural*Treatment	58.143	7.820	0.760
	Semi-natural areas	2000	Intercept + Semi-natural+ Treatment + Semi-natural*Treatment	59.684	9.361	0.730
Plant damage	Cropland	500	Intercept + Cropland + Treatment + Cropland *Treatment	257.881	7.465	0.638
	Cropland	1000	Intercept + Cropland + Treatment + Cropland *Treatment	256.400	5.984	0.690
	**Cropland**	**2000**	**Intercept + Cropland + Treatment + Cropland *Treatment**	**250.417**	**0**	**0.738**
	Semi-natural areas	500	Intercept + Semi-natural+ Treatment + Semi-natural*Treatment	274.720	24.303	0.656
	Semi-natural areas	1000	Intercept + Semi-natural+ Treatment + Semi-natural*Treatment	274.635	24.218	0.627
	Semi-natural areas	2000	Intercept + Semi-natural+ Treatment + Semi-natural*Treatment	272.128	21.711	0.637
Crop biomass	Cropland	500	Intercept + Cropland + Treatment + Cropland *Treatment	341.324	1.885	0.923
	Cropland	1000	Intercept + Cropland + Treatment + Cropland *Treatment	345.487	6.048	0.916
	**Cropland**	**2000**	**Intercept + Cropland + Treatment + Cropland *Treatment**	**339.440**	**0**	**0.926**
	Semi-natural areas	500	Intercept + Semi-natural+ Treatment + Semi-natural*Treatment	346.151	6.712	0.915
	Semi-natural areas	1000	Intercept + Semi-natural+ Treatment + Semi-natural*Treatment	346.367	6.928	0.915
	Semi-natural areas	2000	Intercept + Semi-natural+ Treatment + Semi-natural*Treatment	343.703	4.263	0.913
Predation on sentinel larvae	Cropland	500	Intercept + Cropland + Treatment	310.208	1.749	0.523
	Cropland	1000	Intercept + Cropland + Treatment	310.163	1.703	0.523
	Cropland	2000	Intercept + Cropland + Treatment	309.700	1.240	0.523
	Semi-natural areas	500	Intercept + Semi-natural+ Treatment	309.793	1.333	0.517
	Semi-natural areas	1000	Intercept + Semi-natural+ Treatment	308.594	0.134	0.525
	**Semi-natural areas**	**2000**	**Intercept + Semi-natural+ Treatment**	**308.460**	**0**	**0.519**

Predation on sentinel eggs	Cropland	500	Intercept + Cropland + Treatment + Cropland *Treatment	228.673	2.966	0.528
	Cropland	1000	Intercept + Cropland + Treatment + Cropland *Treatment	227.791	2.084	0.528
	Cropland	2000	Intercept + Cropland + Treatment + Cropland *Treatment	226.908	1.202	0.554
	Semi-natural areas	500	Intercept + Semi-natural+ Treatment + Semi-natural*Treatment	227.561	1.854	0.570
	Semi-natural areas	1000	Intercept + Semi-natural+ Treatment + Semi-natural*Treatment	227.748	2.041	0.543
	**Semi-natural areas**	**2000**	**Intercept + Semi-natural+ Treatment + Semi-natural*Treatment**	**225.707**	**0**	**0.584**
Ground-dwelling predators	Cropland	500	Intercept + Cropland + Treatment	271.105	3.133	0.720
	Cropland	1000	Intercept + Cropland + Treatment	270.958	2.987	0.701
	**Cropland**	**2000**	**Intercept + Cropland + Treatment**	**267.972**	**0**	**0.743**
	Semi-natural areas	500	Intercept + Semi-natural+ Treatment	273.531	5.559	0.723
	Semi-natural areas	1000	Intercept + Semi-natural+ Treatment	273.324	5.352	0.717
	Semi-natural areas	2000	Intercept + Semi-natural+ Treatment	271.174	3.203	0.737
Parasitoids	Cropland	500	Intercept + Cropland + Treatment + Cropland *Treatment	34.934	6.786	0.920
	Cropland	1000	Intercept + Cropland + Treatment + Cropland *Treatment	30.626	2.478	0.924
	Cropland	2000	Intercept + Cropland + Treatment + Cropland *Treatment	31.580	3.431	0.914
	Semi-natural areas	500	Intercept + Semi-natural+ Treatment + Semi-natural*Treatment	33.199	5.050	0.927
	**Semi-natural areas**	**1000**	**Intercept + Semi-natural+ Treatment + Semi-natural*Treatment**	**28.149**	**0**	**0.937**
	Semi-natural areas	2000	Intercept + Semi-natural+ Treatment + Semi-natural*Treatment	32.521	4.373	0.921
Foliar-foraging predators	Cropland	500	Intercept + Cropland + Treatment + Cropland *Treatment	437.467	2.332	0.581
	Cropland	1000	Intercept + Cropland + Treatment + Cropland *Treatment	438.696	3.561	0.576
	Cropland	2000	Intercept + Cropland + Treatment + Cropland *Treatment	438.556	3.422	0.576
	**Semi-natural areas**	**500**	**Intercept + Semi-natural+ Treatment + Semi-natural*Treatment**	**435.135**	**0**	**0.620**
	Semi-natural areas	1000	Intercept + Semi-natural+ Treatment + Semi-natural*Treatment	436.075	0.941	0.615
	Semi-natural areas	2000	Intercept + Semi-natural+ Treatment + Semi-natural*Treatment	435.922	0.787	0.607

1. The marginal R^2 values for each model (variance explained by both the fixed and random factors) were calculated using the methods detailed in Nakagawa and Schielzeth (2013).

Supplementary Table S2. Results of Mantel test[1] of spatial autocorrelation in the residuals of the final models

Response variable / landscape variable	Monte-Carlo test Observation	P-value
Lepidoptera abundance / Cropland 1000 m	-0.021	0.782
Plant Damage / Cropland 2000 m	-0.061	0.999
Crop biomass / Cropland 2000 m	-0.023	0.797
Predation on sentinel larvae/ Semi-natural areas 2000 m	0.002	0.415
Predation on sentinel eggs/ Semi-natural areas 2000 m	-0.026	0.805
Ground-dwelling predators / Cropland 1000 m	-0.028	0.818
Parasitoids / Semi-natural areas 1000 m	-0.015	0.624
Foliar-foraging predators/ Semi-natural areas 500 m	-0.003	0.477

1. All final models were tested for spatial autocorrelation in the residuals using the mantel test from the package ade4 (Dray & Dufour 2007).

Effects of augmentative predator releases on final crop yield

For the purposes of this paper, we draw distinction between marketable crop yield and crop biomass. Marketable crop yield of cabbage is a result of both the harvested head weight and the cosmetic injury to the head. Crop biomass, on the other hand, is an indicator of plant productivity, which is significantly correlated with head weight (Pearson's $r = -0.58$, $P = 0.005$). but does not account for the quality component. In fact, although high levels of lepidopteran defoliation consistently increase feeding injury of cabbage plants (i.e., plant damage was significantly correlated with the mean abundance of lepidopteran larvae, Pearson's $r = 0.33$, $P = 0.002$), they may not always affect harvested head weight due to the ability of brassica crops to tolerate relatively high levels of defoliation without significantly affecting final weight (Burkness *et al.* 2005, Liu *et al*., 2004). For this reason, we evaluated the potential effects of augmentative biocontrol on final crop yield using both quantity (i.e., marketable cabbage head weight) and quality (i.e., cabbage head damage) measures (Supplementary Fig. S1).

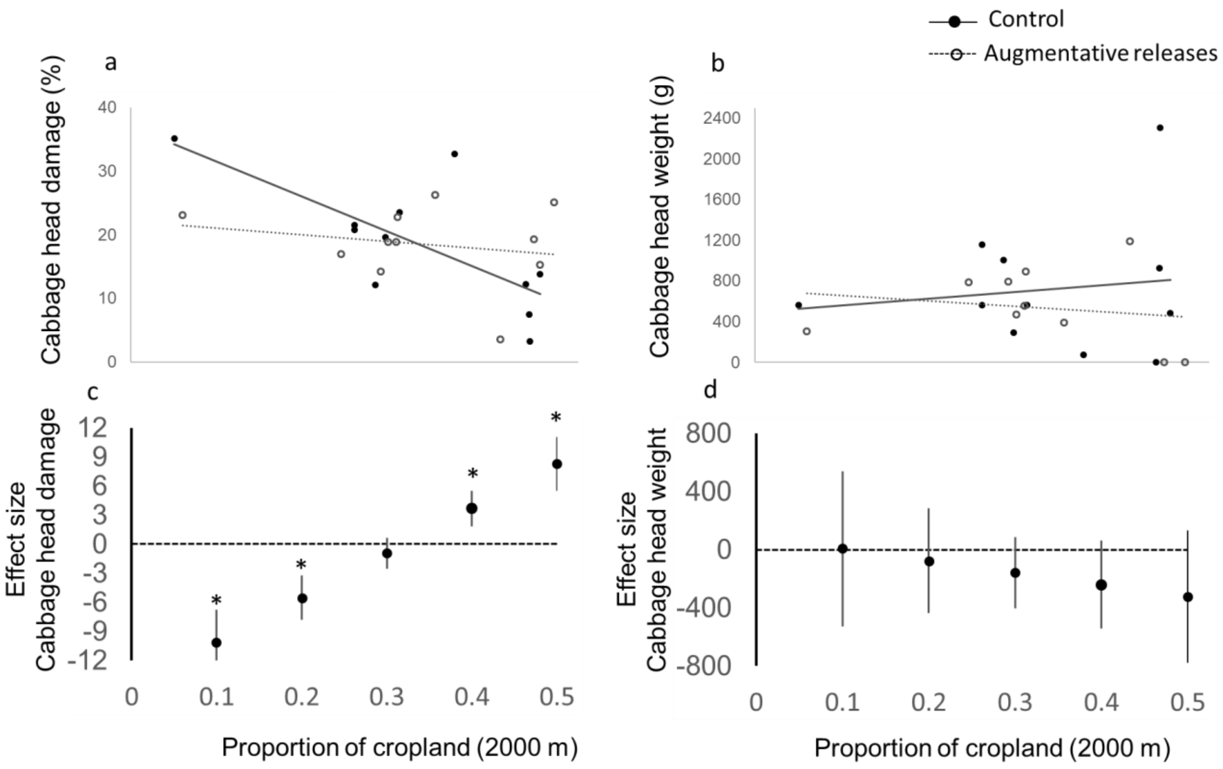

Supplementary Fig. S1. The effect of augmentative releases of predators on final crop yield based on (a) quality (i.e., cabbage head damage) and (b) quantity measures (i.e., cabbage head weight) in landscapes of varying complexity. Predicted responses for the control (solid lines) and augmentative release (dashed lines) treatments are calculated from the set of best supported linear mixed-effects models (lme4). Effects of the interactions between treatment and landscape complexity were significant ($P < 0.05$) for crop yield quality, but not for crop yield quantity. In the top Figures (a and b) every point represents the mean treatment value in a given experimental plot. The bottom figs. (c and d) are effect sizes (mean ± 95 % CI) for crop yield quality (c) and crop yield quantity (d) based on the difference in the marginal means between plots with and without predator releases across the landscape complexity gradient. A positive effect size indicates that the mean of the predator plots is larger than the mean of control plots, while a negative effect size indicates a higher control mean. Pairwise comparisons were individually calculated at even intervals across the landscape complexity gradient. Asterisks denote effect sizes that are significantly different from zero ($P < 0.05$). Summary statistics of the LMER models used to estimate marginal means and confidence intervals are available in Supplementary Table S3.

Supplementary Table S3. Statistical models for the effects of landscape composition and potential interactions with predator releases on both final crop yield quality (i.e., cabbage head damage) and quantity (i.e., cabbage head weight). Statistical models were used to estimate mean and 95% CI of effect sizes for landscape effects and potential interactions with predator releases (Supplementary Fig. S1.). Dashed lines represent interaction terms not included in the final models because they were not significant (P > 0.05). Boldface text indicates significant relationships (P < 0.05).

Response variables and predictors	d.f.[1]	F[1]	P-value[1]
Crop yield quality (i.e., cabbage head damage)			
Cropland (2000 m)	1, 29.898	2.823	0.103
Treatment (control and predator releases)	1, 32.556	16.287	**< 0.001**
Interaction (cropland x treatment)	1, 32.517	18.995	**< 0.001**
Crop yield quantity (i.e., cabbage head weight)			
Cropland (2000 m)	1, 2182.4	0.233	0.629
Treatment (control and predator releases)	1, 3137.8	1.196	0.274
Interaction (cropland x treatment)	-----	-----	-----

1. The statistical significance of fixed effects and interaction terms were estimated using mixed-effect models (lmer) interpreted with a Satterthwaite approximation (Kuznetsova *et al*. 2017, Luke 2017).

References

Burkness E., Gingera G.J. & Hutchison W. D. 2005. Impact of simulated insect defoliation and timing of injury on cabbage yield in Minnesota. Great Lakes Entomologist **38**, 1-12 (2005).

Dray, S. & Dufour. A. B. The ade4 package: Implementing the duality diagram for ecologists. J. Stat. Softw. **22**, 1–20 (2007).

Kuznetsova, A., Brockhoff, P. B. & Christensen, R. H. B. lmerTest Package: Tests in Linear Mixed Effects Models. *J. Stat. Softw*. **82**, (2017).

Liu S.-S. Shi Z.-H. Guo S.-J. Chen Y.-N. Zhang G.-M. Lu L.-F. Wang D.-S. Deuter P. Zalucki M. P. Improvement of crucifer IPM in the Changjiang River Valley, China: from research to practice, pp. 61–66. In Endersby N. M. Ridlands P. M. [eds.], The Management of Diamondback Moth and Other Crucifer Pests: Proceedings of the 4th International Workshop, 26–29 November 2001, Melbourne, Australia. The Regional Institute, Gosford, Australia (2004).

Luke, S. G. Evaluating significance in linear mixed-effects models in R. *Behav. Res. Methods* **49**, 1494–1502 (2017).

Nakagawa, S. & Schielzeth, H. A general and simple method for obtaining R^2 from generalized linear mixed-effects models. *Methods Ecol. Evol.*, **4**, 133–142 (2013).
